# A Phase 1 study of ADI‐PEG20 (pegargiminase) combined with cisplatin and pemetrexed in ASS1‐negative metastatic uveal melanoma

**DOI:** 10.1111/pcmr.13042

**Published:** 2022-05-16

**Authors:** Pui Ying Chan, Melissa M. Phillips, Stephen Ellis, Amanda Johnston, Xiaoxing Feng, Amit Arora, Gordon Hay, Victoria M. L. Cohen, Mandeep S. Sagoo, John S. Bomalaski, Michael T. Sheaff, Peter W. Szlosarek

**Affiliations:** ^1^ Department of Medical Oncology St Bartholomew's Hospital, Barts Health NHS Trust London UK; ^2^ Wellcome Sanger Institute, Hinxton Cambridgeshire UK; ^3^ Polaris Pharmaceuticals Inc San Diego California USA; ^4^ Department of Ocular Oncology, Moorfields Eye Hospital Moorfields Eye Hospital NHS Foundation Trust London UK; ^5^ NIHR Biomedical Research Centre for Ophthalmology at Moorfields Eye Hospital and University College London Institute of Ophthalmology London UK; ^6^ Department of Histopathology, Royal London Hospital Barts Health NHS Trust London UK; ^7^ Centre for Cancer Biomarkers and Biotherapeutics, Barts Cancer Institute Queen Mary University of London London UK

**Keywords:** ADI‐PEG20, arginine auxotrophy, ASS1, cisplatin, pemetrexed, uveal melanoma

## Abstract

Metastatic uveal melanoma (UM) is a devastating disease with few treatment options. We evaluated the safety, tolerability and preliminary activity of arginine depletion using pegylated arginine deiminase **(**ADI‐PEG20; pegargiminase) combined with pemetrexed (Pem) and cisplatin (Cis) chemotherapy in a phase 1 dose‐expansion study of patients with argininosuccinate synthetase (ASS1)‐deficient metastatic UM. Eligible patients received up to six cycles of Pem (500 mg/m^2^) and Cis (75 mg/m^2^) every 3 weeks plus weekly intramuscular ADI (36 mg/m^2^), followed by maintenance ADI until progression (NCT02029690). Ten of fourteen ASS1‐deficient patients with UM liver metastases and a median of one line of prior immunotherapy received ADIPemCis. Only one ≥ grade 3 adverse event of febrile neutropenia was reported. Seven patients had stable disease with a median progression‐free survival of 3.0 months (range, 1.3–8.1) and a median overall survival of 11.5 months (range, 3.2–36.9). Despite anti‐ADI‐PEG20 antibody emergence, plasma arginine concentrations remained suppressed by 18 weeks with a reciprocal increase in plasma citrulline. Tumour rebiopsies at progression revealed ASS1 re‐expression as an escape mechanism. ADIPemCis was well tolerated with modest disease stabilisation in metastatic UM. Further investigation of arginine deprivation is indicated in UM including combinations with immune checkpoint blockade and additional anti‐metabolite strategies.

**Significance**

ADI‐PEG20 monotherapy has clinical activity in multiple ASS1‐deficient tumours including melanoma. In this biomarker‐directed phase 1 study, ADI‐PEG20 was administered with platinum‐based chemotherapy in a cohort of largely immunorefractory metastatic uveal melanoma patients. Treatment was well tolerated with clinical efficacy, evidenced by disease stabilisation and median progression‐free survival and overall survival of 3.0 and 11.5 months, respectively. ASS1 re‐expression was identified at disease progression. Based on this work, further early phase trials optimising ADI‐PEG20 activity are currently underway in metastatic uveal melanoma including with combination immunotherapy.

## 1 INTRODUCTION

Uveal melanoma (UM) has a unique biology characterised by low programmed cell death ligand‐1 (PD‐L1) expression, low mutational burden and liver‐centric metastases with hepatic failure as the main mode of death (Javed et al., 2016; Royer‐Bertrand et al., 2016). As such, the durable clinical efficacy of anti‐programmed death 1 receptor (PD‐1) and anti‐cytotoxic T‐lymphocyte antigen 4 (CTLA‐4) inhibition in cutaneous melanoma is rarely observed in this patient population (Algazi et al., 2016; Chan et al., 2017; Karydis et al., 2016; Zimmer et al., 2015). Median overall survival (OS) following immune checkpoint blockade remains low across multiple studies at 6–9 months (Algazi et al., 2016; Zimmer et al., 2015). With approximately 50% of patients developing liver metastases following radical therapy to the primary tumour, new treatment strategies are urgently needed.

Arginine is a semi‐essential amino acid that promotes tumour growth. It is key to numerous biosynthetic pathways for production of proteins, polyamines, nitric oxide and the amino acids proline and glutamate. Tumours that are deficient in the urea cycle enzyme argininosuccinate synthetase (ASS1) are unable to biosynthesise argininosuccinate derived from citrulline and aspartate and the direct precursor for arginine. Termed arginine auxotrophy, ASS1‐deficient tumours depend on the direct uptake of exogenous arginine for growth (Keshet et al., 2018).

ASS1 deficiency is observed at high frequency in numerous chemoresistant solid tumours including metastatic melanoma (Dillon et al., 2004). ASS1 deficiency has been associated with accelerated tumourigenesis and more aggressive cancers, conferring worse survival outcomes (Huang et al., 2013). ASS1‐deficient tumours exhibit increased proliferation as a result of diversion of aspartate towards enhanced nucleotide synthesis (Rabinovich et al., 2015). Intratumoural ASS1 loss has been employed as a biomarker selecting for sensitivity to arginine deprivation therapy. A therapeutic window exists for using arginine‐depleting agents to selectively induce cell death in ASS1‐deficient tumours, whilst maintaining normal cells that replete arginine through endogenous conversion of citrulline.

Arginine deiminase is a mycoplasma‐derived enzyme that irreversibly catalyses the hydrolysis of arginine to citrulline and ammonia. Single agent recombinant, pegylated ADI‐PEG20 (ADI; pegargiminase) has efficacy with low toxicity in clinical trials in ASS1‐deficient tumours including hepatocellular carcinoma (Glazer et al., 2010), mesothelioma (Szlosarek et al., 2017) and cutaneous melanoma (Ascierto et al., 2005; Feun et al., 2012). In a phase 1/2 study of single‐agent ADI‐PEG20 in melanoma, a high rate of stable disease (SD) was observed in four out of six UM patients, with durable responses in two patients up to 11 months, encouraging further exploration in combination with chemotherapy (Ott et al., 2013).

Administration of ADI‐PEG20 in combination chemotherapy is validated by evidence of potentiation of the cytotoxic effects of folate inhibitors and platinum compounds in ASS1‐deficient tumour cells. Preclinical studies have demonstrated suppression of both de novo pyrimidine synthesis and the pyrimidine salvage pathway with the combination ADI‐PEG20 and pemetrexed (Allen et al., 2013), countering the enhanced pyrimidine synthesis and proliferation of ASS1‐deficient tumours (Rabinovich et al., 2015). Moreover, significantly enhanced anticancer effects of ADI‐PEG20 combined with cisplatin were observed in cell lines and xenograft models of melanoma compared with either agent alone, and attributed to a reduction in DNA repair proteins and alteration of pro‐ and anti‐apoptotic proteins (Savaraj et al., 2015).

In a 3 + 3 + 3 phase 1 dose‐escalation study of patients with ASS1‐deficient thoracic cancers, we demonstrated tolerability and a high disease control rate (DCR) using weekly ADI‐PEG20 at the maximum tolerated dose of 36 mg/m^2^ combined with first‐line pemetrexed and cisplatin (ADIPemCis); no dose‐limiting toxicities were reported, and there were no treatment‐related deaths (Beddowes et al., 2017). Additional expansion cohorts were opened in multiple tumour types including in patients with extensively treated high‐grade glioma in whom significant activity of ADIPemCis was documented (Hall et al., 2019).

This was an open label multicentre expansion cohort of a phase 1 trial of ADIPemCis at the recommended phase 2 dose (RP2D), in patients with histologically proven metastatic UM. The primary objective of the study was to assess safety and toxicity and obtain preliminary estimates of efficacy of the RP2D of ADI‐PEG20 in combination with pemetrexed and cisplatin. Secondary objectives of the study were to determine: (1) progression‐free survival (PFS) and OS, (2) pharmacodynamics and immunogenicity of ADI‐PEG20 in combination with pemetrexed and cisplatin and (3) mechanisms of intrinsic resistance to ADI‐PEG20 by rebiopsying tumours on progression.

## 2 METHODS

Between November 2015 and July 2016, patients at least 18 years old with ASS1‐deficient histologically proven metastatic disease and measurable disease by Response Evaluation Criteria in Solid Tumors (RECIST) version 1.1 were considered eligible if they had adequate haematologic, hepatic and renal function; Eastern Cooperative Oncology Group performance status 0 or 1; and a life expectancy of at least 3 months. Prior immunotherapy, but not chemotherapy, was permitted. Deficiency was determined semiquantitatively by absent or reduced ASS1 immunohistochemical staining (0 or 1 plus staining) in >50% of tumour cells from tissue specimens, as described previously (Beddowes et al., 2017). ASS1 immunohistochemical scoring was performed independently by an accredited histopathologist. Patients were excluded from the study if they had received prior ADI‐PEG20 treatment; prior targeted therapy within 4 weeks of study entry; ongoing toxic manifestations from previous treatments; symptomatic brain or spinal cord metastases; history or co‐existence of another primary cancer; recent major surgery; therapeutic anticoagulation; or known allergies to the study drugs.

Intramuscular ADI‐PEG20 (36 mg/m^2^) was administered once weekly with intravenous pemetrexed (500 mg/m^2^) and cisplatin (75 mg/m^2^) once every 3 weeks up to a maximum of 18 weeks (six cycles). Patients with disease control were allowed to continue receiving ADI‐PEG20 monotherapy beyond six cycles until disease progression. Patients who had progressed whilst on study were offered to consent to having an additional biopsy for immunohistochemical assessment of ASS1 status.

Computed tomographic (CT) or magnetic resonance imaging (MRI) scans were performed at baseline and scheduled every 6 weeks during ADIPemCis treatment, and every 8 weeks during ADI‐PEG20 monotherapy. The same modality of imaging was used throughout the study for each patient. Radiological tumour response was assessed according to RECIST 1.1. Best overall response, defined as the best response recorded from the start of treatment until the end of treatment, was used as the primary efficacy endpoint. PFS and OS, response rate and disease control rate (DCR) were used as secondary efficacy endpoints. Response rate and DCR were determined radiologically, according to RECIST 1.1. PFS events were defined as progression, according to RECIST 1.1 or death from any cause. OS events were defined as death from any cause. Patients were contacted every 3 months following their end of treatment assessment until death to determine survival status. All endpoints were measured from the date from first treatment with ADI‐PEG20.

To perform pharmacodynamics assays, plasma arginine and citrulline levels were measured using liquid chromatography‐mass spectrometry as described previously (Beddowes et al., 2017). Anti‐ADI‐PEG20 antibody titres were measured using an enzyme‐linked immunosorbent assay as described previously (Beddowes et al., 2017).

The study was approved by the Leeds East (Yorkshire and The Humber) ethical review board. The study was conducted in accordance with the Declaration of Helsinki and Good Clinical Practice guidelines. All patients provided written informed consent.

## 3 RESULTS

All 14 screened patients were negative for ASS1 expression. Two patients were ineligible (severe aortic stenosis; and a concurrent cancer diagnosis of sarcomatoid malignant pleural mesothelioma), and two patients opted for immune checkpoint blockade. Two treatment naive patients and eight patients previously exposed to immunotherapy were enrolled into the study (median 1 prior systemic treatment, range 0–5). All ten patients had liver metastases; five patients had additional extrahepatic metastases. At baseline, four patients had an Eastern Collaborative Oncology Group (ECOG) performance score of zero and six patients had an ECOG performance score of 1. Individual patient characteristics at baseline are shown in Table 1. Patients received a median of four cycles of chemotherapy, and 70% of patients required a dose reduction of pemetrexed and/or cisplatin at some point during their treatment. The median duration of treatment with ADI‐PEG20 was 12.4 weeks (range, 7–36 weeks).

**TABLE 1:** Baseline characteristics of patients receiving ADIPemCis

Patient	Age at study enrolment	Gender	Baseline LDH (240–480 U/L)	Date of primary tumour diganosis	Site of primary tumour	Treatment for primary tumour	Date of metastatic disease	Site(s) of metastatic disease	Prior treatment for metastatic disease	Baseline plasma arginine (μM)	Baseline plasma citrulline (μM)	Baseline anti‐ADI‐PEG20 antibody titre	Treatment post‐progression
A	67	M	551	18/06/09	Choroidal	Enucleation	17/02/15	Liver	Ipilimumab, interferon‐alpha, pembrolizumab	43.4	31.3	1	Palliative care
B	37	F	294	21/08/14	Choroidal	Enucleation	11/01/16	Liver	Ipilimumab	22.3	17.8	0	Pembrolizumab
C	60	M	344	01/10/10	Choroidal	Enucleation	29/01/16	Liver, lung, bone, retroperitoneal, adrenal	Ipilimumab	81.8	38.2	0	Pembrolizumab, temozolomide
D	61	M	420	08/03/13	Choroidal	Enucleation	06/05/15	Liver	Ipilimumab	56.4	34.8	2	Pembrolizumab
E	35	F	5052	19/04/12	Choroidal	Plaque brachytherapy	18/04/16	Liver	Ipilimumab, pembrolizumab, AKT inhibitor*, interferon‐alpha, selumetinib with vistusertib*	42.2	10.5	2	Palliative care
F	53	F	1248	13/03/14	Choroidal	Plaque brachytherapy	13/04/14	Liver	None	n/a	n/a	n/a	Ipilimumab, pembrolizumab
G	41	F	1696	01/01/13	Choroidal	Plaque brachytherapy	01/05/15	Liver, lung	Ipilimumab, pembrolizumab, hepatic melphalan	57.9	26.8	0	Palliative care
H	73	F	536	14/06/12	Choroidal	Plaque brachytherapy followed by enucleation	30/10/15	Liver, lung, bone	None	99.1	34.2	0	Pembrolizumab, ipilimumab, temozolomide
I	79	F	860	08/12/11	Choroidal	Plaque brachytherapy	09/04/15	Liver, bone, retroperitoneal	Ipilimumab, interferon‐alpha	109	32.9	0	Pembrolizumab, temozolomide
J	62	F	1077	06/02/09	Choroidal	Plaque brachytherapy	17/07/15	Liver, retroperitoneal, subcutaneous	Ipilimumab	60.4	18.7	2	Pembrolizumab, temozolomide

*Note*: Treatments received through phase 1 clinical trials are indicated with an asterisk.

Abbreviations: ADI‐PEG20, pegylated arginine deiminase; F, female; LDH, lactate dehydrogenase; M, male.

Treatment was well tolerated overall and summarised in Table 2. 30% of patients experienced no adverse events (AEs) per Common Terminology Criteria for Adverse Events (NCI CTCAE) version 4.03. Five patients (50%) experienced grade 1 or 2 AEs. Those deemed possibly or probably related to ADI‐PEG20 were rash, skin ulceration and lethargy. At least one grade 3 or 4 AE was observed in five patients (50%); the most common grade 3 or 4 AE was neutropenia in four patients (40%), followed by thrombocytopenia in two patients (20%). No grade 3 or 4 AEs were related to ADI‐PEG20 treatment. There were no treatment‐related deaths.

**TABLE 2:** Number of patients with reported adverse events based on CTCAE v4.03 for ADIPemCis

	Total number of patients with AE		Number of patients with AE possibly or probably related to ADI‐PEG20	
Adverse event	Grade 1–2	Grade 3–4	Grade 1–2	Grade 3–4
Alopecia	1			
Blood bilirubin increase	1			
Musculoskeletal Pain	1			
Renal impairment	1			
Skin ulceration	1		1	
Lethargy	2		2	
Rash	2		2	
Hepatobiliary disorder		1		
Portal vein thrombosis		1		
Neutropenia		4		
Neutropenic sepsis		1		
Thrombocytopenia	1	2		

Abbreviations: ADI‐PEG, pegylated arginine deiminase; AE, adverse event; CTCAE, Common Terminology Criteria for Adverse Events.

**FIGURE 1:**
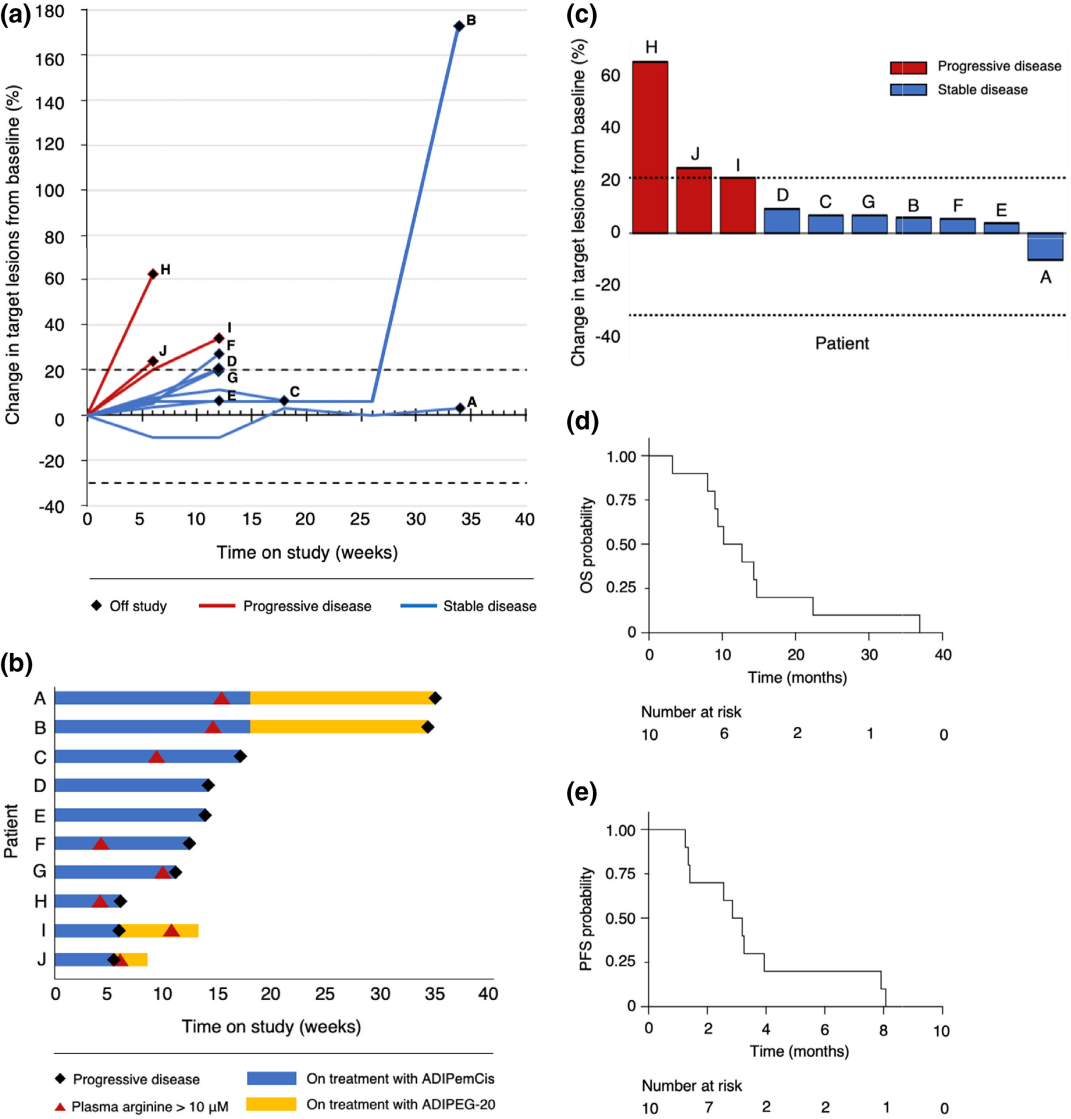
(a) Spider plot showing the percentage change in the sum of the target lesion diameters over time. (b) Swimmer plot showing time on treatment with ADIPemCis and ADI‐PEG20, time to raised plasma arginine levels and time to progression. (c) Waterfall plot showing the best percentage change in the sum of the target lesion diameters. (d) Kaplan–Meier analysis of overall survival (OS) in all patients. (e) Kaplan–Meier analysis of progression‐free survival (PFS) in all patients

All patients had evaluable disease at the first response assessment on week six. There were no objective responses. The best overall response was SD in seven out of ten patients; the best DCR was 70% at Week 12. The remaining three patients had progressive disease at the first response assessment. The median PFS was 3.0 months (range, 1.3–8.1 months), and the median OS was 11.5 months (range, 3.2–36.9 months). Notably, SD lasting longer than 6 months was observed in two pre‐treated patients who received one and three prior lines of systemic immunotherapy (Figure 1).

**FIGURE 2:**
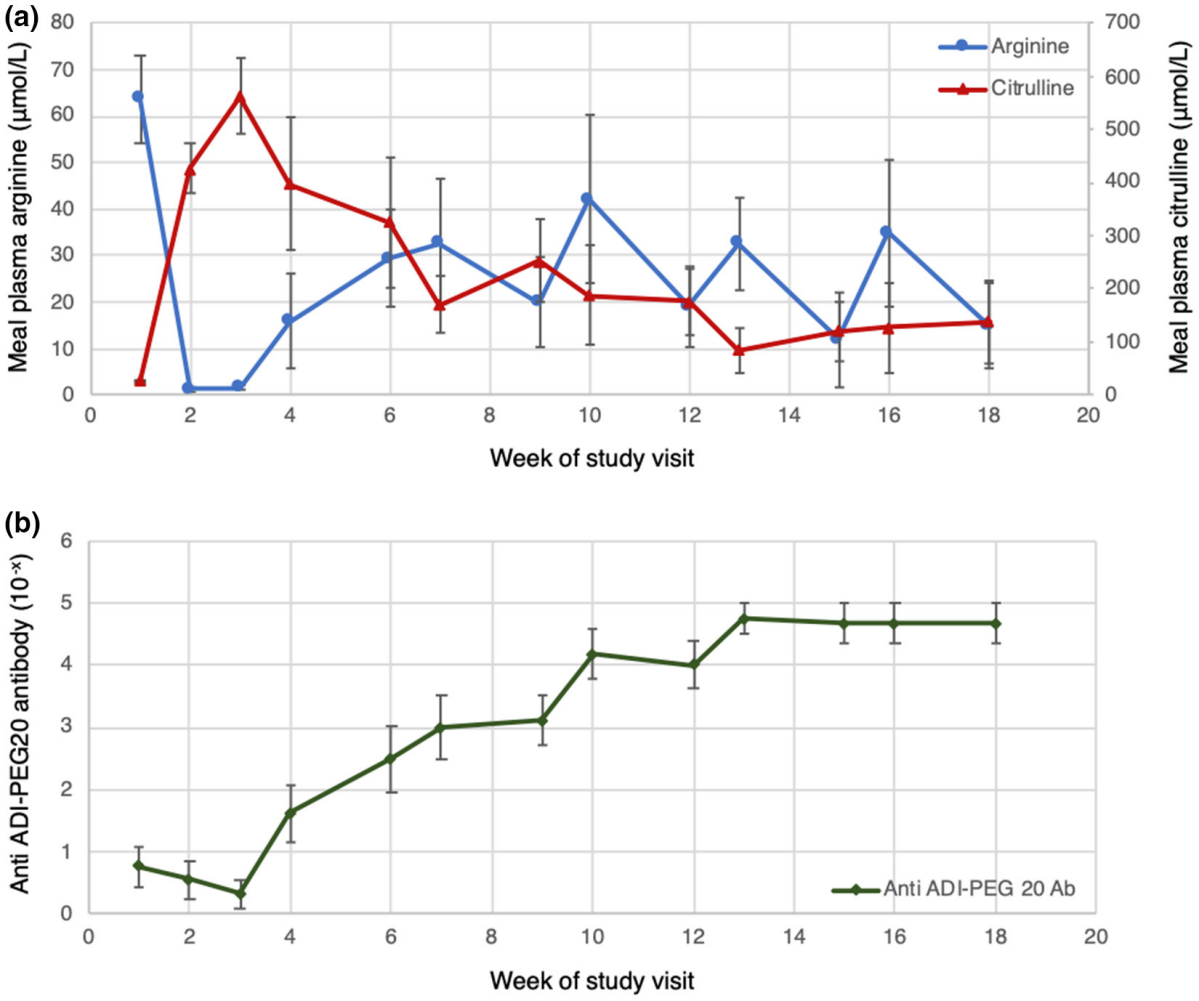
(a) Mean plasma concentration of arginine and citrulline at each week of treatment for the study population. (b) Mean plasma anti‐ADI‐PEG20 antibody titres at each week of treatment for the study population. Error bars indicate the standard error of the mean (SEM). Ab, antibody; ADI‐PEG20, pegylated arginine deiminase

**FIGURE 3:**
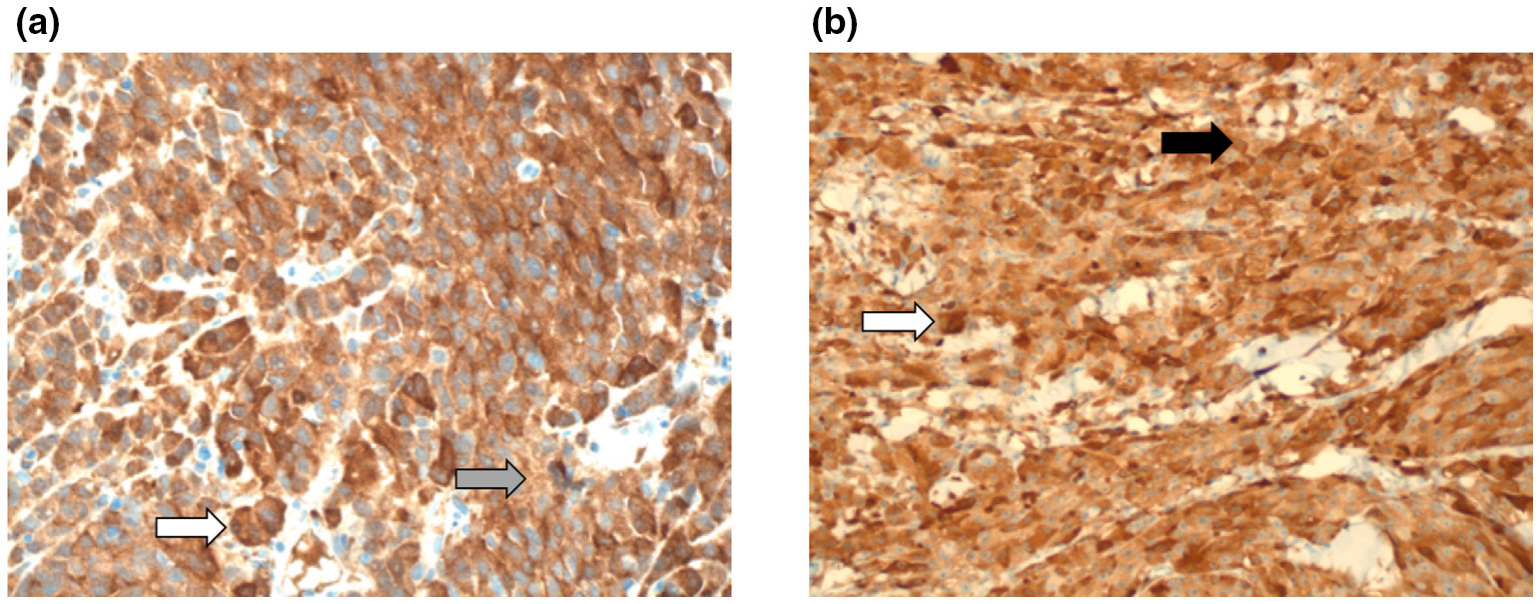
Representative ASS1 tumoural expression at baseline (a, grey arrow, liver deposit with weak staining intensity) and at progression (b, black arrow, skin deposit with moderate‐strong staining intensity) in a patient with metastatic UM (×400). The larger strongly ASS1 expressing cells in the baseline and progression biopsies are consistent with macrophages (i.e. melanophages, white arrows)

Plasma arginine concentrations were depleted following the first dose of ADI‐PEG20 in all patients during the first cycle of ADIPemCis. Anti‐ADI‐PEG20 antibody titres rose gradually at week four and plateaued by Week 13. Despite the emergence of anti‐ADI‐PEG20 antibodies, plasma arginine concentrations remained suppressed at one‐third of baseline levels by Week 18 of triplet therapy with a reciprocal increase in plasma citrulline concentration (Figure 2). Tumour tissue from biopsies at progression was available from two patients, both with cutaneous metastases. ASS1 expression was assessed immunohistochemically and was re‐expressed in both patients (Figure 3).

## 4 DISCUSSION

In the current biomarker‐directed study of ADI‐PEG20 with pemetrexed and cisplatin, we provide evidence for safety and preliminary activity of ADI‐containing chemotherapy in patients with metastatic UM. Despite a lack of objective responses, treatment was well tolerated with a median PFS and OS of 3.0 and 11.5 months, respectively, in a predominantly CTLA‐4 inhibitor ipilimumab pre‐treated population.

There are currently no approved systemic chemotherapeutic agents that have improved survival specifically for patients with metastatic UM in a phase 3 study. Since metastatic UM are globally deficient in ASS1 and display sensitivity to ADI‐PEG20, this presents a good opportunity for developing metabolic therapies based on arginine deprivation. It is noteworthy that the 50% OS rate at 1 year in our largely pre‐treated cohort of metastatic UM patients (70% of whom had raised LDH levels), compares favourably with previous reports of 20% in the treatment naïve setting (Diener‐West et al., 2005). Specifically, prolonged SD of 7.9 and 8.1 months was observed in two patients who had progressed on pembrolizumab and/or ipilimumab.

Consistent with the dose‐escalation cohort, arginine levels remained suppressed for a longer duration with the combination ADIPemCis compared with ADI‐PEG20 monotherapy (Beddowes et al., 2017; Szlosarek et al., 2017; Ott et al., 2013). The observed duration of arginine suppression and reciprocal increase in plasma citrulline levels was shorter compared with patients in the dose‐escalation thoracic study receiving ADIPemCis; however, this may be confounded by the fewer number of patients analysed due to earlier disease progression (Beddowes et al., 2017). For the majority of patients in this study, normalising arginine/citrulline levels correlated with resistance to treatment. However, in patients where arginine levels remained suppressed and citrulline levels remained high even at the point of progression, antibody titres were raised. We note that patients A and B with the longest PFS continued to respond to therapy with stable disease for a further 5 months after rising antibody titres and normalised arginine/citrulline levels. Importantly, arginine/citrulline levels were measured once a week just before dosing with ADI‐PEG20 thus it is possible that intermittent and short‐lived reductions of arginine—and spikes in citrulline—levels occurred, but that this pharmacodynamic effect was not captured within the study design. Hence, continued treatment has been permitted in studies of ADI‐PEG20, despite normalisation of arginine and citrulline levels.

We sought to understand the mechanism of ASS1 repression by interrogating the ASS1 methylation status of the UM biopsies using the Infinium methylation EPIC (450 K) array. Sufficient tumour DNA at baseline was available for one patient only revealing modest methylation at the ASS1 promoter (0.46, data not shown). This is consistent with previous data on epigenetic silencing of ASS1 as a mechanism mediating arginine auxotrophy in cancer cell lines and primary tumours (Allen et al., 2013; Szlosarek et al., 2017). Moreover, upregulation of ASS1 appears to be a key resistance mechanism to ADI‐PEG20 in UM as noted in two patients rebiopsied on progression. Although ASS1 demethylation may be involved, as noted in an earlier study in cutaneous melanoma, c‐myc may be also a potential driver of ASS1 upregulation under arginine depletion in UM (Feun et al., 2012). Specifically, c‐myc abnormalities have been reported in 70% of uveal melanomas with amplification a frequent event in association with monosomy 3 (Parrella et al., 2001).

The 70% DCR is encouraging in UM and validates a smaller ADI‐PEG20 monotherapy study revealing a DCR of 67% in six patients, of whom two had SD for 7.5 and 11.2 months with a median PFS of 3.7 months (Ott et al., 2013). Furthermore, a recent dose‐expansion study of doublet therapy with ADI‐PEG20 (36 mg/m^2^ intramuscular weekly) and cisplatin (30 mg/m^2^ intravenous on Days 1, 8 and 15 on a monthly cycle) in multiple tumour types also revealed a DCR of 67% and a median OS of approximately 12 months in metastatic UM (*n* = 9) (Yao et al., 2021). Thus, overall the addition of cisplatin and pemetrexed do not biochemically potentiate ADI‐PEG20 in the context of UM in contrast to thoracic cancers (Beddowes et al., 2017). Furthermore, the median OS data are encouraging in light of previous chemotherapy studies in UM, in which platinum‐based anti‐metabolite combinations have yielded inferior results. For instance, the median OS for the triplet drug combination of cisplatin, treosulfan and gemcitabine was 6 months only in relapsed UM (Atzpodien et al., 2008). Radioembolisation with yttrium‐90 microspheres and intravenous cisplatin yielded a median OS of 10 months in a largely treatment naïve population (Arulananda et al., 2019). Although antifolates such as pemetrexed have not been tested previously in UM, and as identified in this study, lack efficacy in UM, further traction may be gained instead by exploring arginine deprivation with taxanes. Both docetaxel and paclitaxel induce disease stabilisation in UM and are potentiated by ADI‐PEG20 in patients with various cancers (Atzpodien et al., 2008; Arulananda et al., 2019; Bhatia et al., 2012; Lee et al., 2015; Lowery et al., 2017; Tomlinson et al., 2015).

Notably, ADI‐PEG20 has been reported to increase pyrimidine salvage in melanoma cell lines in vivo via upregulation of thymidine kinase 1, highlighting differential biology compared with arginine tumour auxotrophs of epithelial origin (Stelter et al., 2013). This further highlights the importance of tissue lineage as a critical determinant of ADI‐PEG20 sensitivity in arginine‐dependent cancers as assessed by ASS1 immunohistochemistry alone. Thus, analysis of upstream gene regulators of ASS1 status is warranted including p53 whose inactivation leads to increased sensitivity to arginine deprivation (Harbour, 2012; Miyamoto et al., 2017). Moreover, the modulation of AKT by ASS1 is an active area for drug targeting in cutaneous and uveal melanoma (Krantz et al., 2017; Long et al., 2013). In contrast, although BAP1 loss was shown recently to induce ASS1 in malignant mesothelioma cell lines with an inverse correlation in clinical samples, the same relationship is not apparent in uveal melanoma (Barnett et al., 2021). Indeed, we identified absence of ASS1 tumoural expression in all 14 metastatic biopsy samples in the present study. Additionally, we reported on widespread deficiency of ASS1 in a majority (74%) of 102 primary choroidal and ciliary body melanomas, despite the known 60–80% loss of BAP1 in uveal melanoma (Harbour et al., 2010; Khadeir et al., 2005; Shah et al., 2013). Interestingly, we identified that in the primary cases with focal ASS1 expression (5%–30%) the majority of cells with the highest expression were in fact CD68‐positive macrophages (or melanophages) and not melanoma cells (Khadeir et al., 2015).

Expanding work on the role of arginine in the immune system under normal and pathological states has emphasised a central role for the amino acid in cancer progression and therapy (Marti & Reith, 2021). Thus, arginine is essential for T‐cell‐mediated anti‐tumour immune responses, and tumour‐derived arginases have been studied extensively over the last two decades as a mechanism of immunosuppression (Munder, 2009). Moreover, arginase inhibitors in combination with immune checkpoint blockade and arginine supplementation are under investigation in various malignancies (Kazmierczak‐Siedlecka et al., 2020; Miret et al., 2019). This contrasts with work showing that arginine deprivation with ADI‐PEG20 may enhance PD‐1/PD‐L1‐based immune checkpoint blockade via upregulation of PD‐L1 tumoural expression and modulation of a T‐cell infiltrate in aggressive tumour xenograft models including B16 melanoma (Brin et al., 2017). The potential for immunosuppressive effects of ADI‐PEG20 may be mitigated by the ability of T cells to replenish arginine via uptake of citrulline via the cationic amino acid transporter (CAT‐1) (Werner et al., 2017). Similarly, the human pegylated arginase, pegzilarginase is active preclinically in solid tumour mouse models with anti‐PD‐L1 and agonist anti‐OX40 immunotherapy (Badeaux et al., 2021). Indeed, urea cycle dysregulated cancers, with functional loss of ASS1, have been linked to favourable genetic and biochemical signatures associated with increased responsiveness to immune checkpoint blockade (Lee et al., 2018). Arginine deprivation with pegylated arginase has also reported clinical activity in melanoma refractory to immunotherapy (De Santo et al., 2018).

Recently, ADI‐PEG20 was tested with pembrolizumab in a phase 1b study in patients with a variety of solid cancers (*n* = 25). Despite the low clinical benefit of anti‐PD‐1 monotherapy with pembrolizumab or nivolumab in metastatic uveal melanoma, the combination was well tolerated with an unexpected increase in neutropenic events (40%) and a partial response rate of 24% in patients with treatment‐refractory cancers (Chang et al., 2021). Moreover, newer combination therapies with immune checkpoint inhibitors have shown moderate responses in this population. Pembrolizumab combined with the HDAC inhibitor entinostat have reported an ORR of 14% in metastatic UM patients, including durable responses (Ny et al., 2021). Ipilimumab combined with nivolumab in metastatic uveal melanoma patients have demonstrated moderate activity with median PFS between 3.0 and 5.5 months and median OS between 12.7 and 19.1 months (Pelster et al., 2021; Piulats et al., 2021). Based on emerging preclinical data for enhancement of immune checkpoint blockade by arginine deprivation in urea cycle dysregulated cancers, and higher objective response rates in UM with dual immune checkpoint inhibition, a phase 1 study of ADI‐PEG20 with CTLA‐4 and PD‐1 combined immune checkpoint blockade has accrued in advanced UM (NCT03922880) for which results are awaited. T‐cell‐directed approaches in combination with arginine deprivation may also be worthy of exploration (Fultang et al., 2020). Indeed, the documented survival benefit of tebentafusp in HLA‐A*02:01–positive metastatic uveal melanoma and the ability of ADI‐PEG20 to generate citrulline and maintain the immune cell compartment provides an opportunity for further development of immunometabolic approaches for UM (Nathan et al., 2021; Werner et al., 2017).

In conclusion, as this study yielded outcomes similar to ADI‐PE20 monotherapy in metastatic UM, our findings support further optimisation of ADI‐PEG20 in combination with alternate systemic therapies in metastatic UM, including immune checkpoint blockade, T‐cell‐directed strategies and novel anti‐metabolite therapeutics.

## CONFLICT OF INTEREST

XF, AJ and JSB are employees of Polaris Pharmaceuticals Inc. MTS has an advisory role with Roche Molecular Diagnostics. PWS has received honoraria from Merck & Co Inc., Merck KGaA, Roche, Bristol‐Myers Squibb and Boehringer Ingelheim. PWS is a recipient of research funding from Polaris Pharmaceuticals Inc. All remaining authors have declared no conflicts of interest.

## Data Availability

The data that support the findings of this study are available from the corresponding author upon reasonable request.
